# Application of large volume injection for sensitive LC-MS/MS analysis of seven artificial sweeteners in surface waters

**DOI:** 10.1016/j.mex.2020.101134

**Published:** 2020-11-07

**Authors:** Jonas Henschel, Heiko Hayen

**Affiliations:** Institute of Inorganic and Analytical Chemistry, University of Münster, Corrensstraße 30, 48149 Münster, Germany

**Keywords:** Surface water analysis;Mixed-mode chromatography;Liquid chromatography-tandem mass spectrometry

## Abstract

The combination of large volume injection and mixed-mode chromatography was performed for direct ultra-trace LC-MS/MS analysis of seven artificial sweeteners with varying physicochemical properties in surface water samples.

• The injection volume was raised from 10 µL to 500 µL, while the overall analysis time was only increased by ≈5 min compared to the initial method.
• Online column head refocusing and concentration of analytes enabled detection in sub-ng L^−1^ concentration range without elaborate sample preparation steps.
• Relative standard deviations <7% despite multiple injection into the loop.

## Graphical abstract

**Figure:**
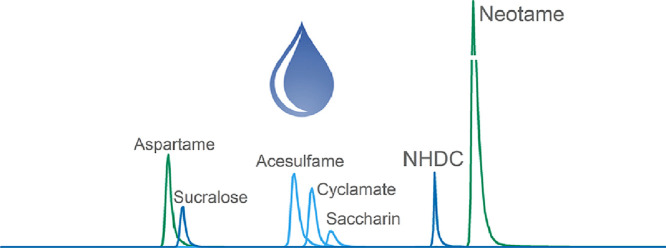

**Abbreviations**
(LVI): large volume injection
(MRM): multiple reaction monitoring
(NHDC): neohesperidin dihydrochalcone
(ACN): acetonitrile
(PTFE): polytetrafluoroethylene
(HILIC): hydrophilic interaction liquid chromatography
(SPE): solid phase extraction
(LOD): limit of detection
(LOQ): limit of quantification

## Specifications Table

**Table:** 

Subject Area	Surface water analysis
More specific subject area	LVI-LC-MS/MS for artificial sweetener analysis
Method name	Not applicable. A new method is presented.
Name and reference of original method	There are no special resources. All experimental details are given in the manuscript to reproduce the method.
Resource availability	Surface water analysis

## Method details

Artificial sweeteners as sugar substitutes have become popular in today's calorie-conscious society. The significant increase in application results in the presence of some persistent compounds in the aquatic environment, detectable in the effluent of waste water plants and even in mineral water [1], [2], [3], [4]. The structural features of artificial sweeteners are highly diverse, and thus complicate the chromatographic separation of multiple compounds with a single technique [5]. Therefore, a method was developed using mixed-mode chromatography on a stationary phase with C18-akyl and anion exchange properties. The separation of (i) three anionic sulfamates (acesulfame, cyclamate, saccharin), (ii) two zwitterionic dipeptides (aspartame, neotame), and (iii) two polar derivates of the natural products sucralose and neohesperidin dihydrochalcone (NHDC) was achieved. Although the use of hydrophilic interaction liquid chromatography (HILIC) columns has proven to be a successful approach for the analysis of polar sweeteners [6,7], the mixed-mode separation showed improved peak shapes and HILIC is not compatible with the direct injection of large volumes when the injection solvent is water, as investigated in detail by Ruta et al. [8].

The simultaneous investigation of these seven partly persistent artificial sweeteners in surface water requires a highly sensitive detection technique, commonly accompanied with a preconcentration strategy. Solid phase extractions (SPE) for analytes with a broad polarity range can be complex as well as costly and time-consuming [9]. Hence, the implementation of a large volume injection (LVI) represents a suitable alternative to extraction techniques. Furthermore, the developed mixed-mode method operated in reversed-phase mode allowing LVIs up to 500 µL. This increase in injected volume combined with a refocusing at the column head improved the limits of detection and quantification to the low and sub-ng L^1^ concentration range. Common LVI issues like analyte breakthrough, peak broadening due to ineffective refocusing at the column head and high matrix stress for the stationary phase were considered during the method development. Consequently, the ultra-trace analysis of artificial sweeteners in surface water without elaborate sample preparation steps was enabled. The combination of mixed-mode chromatography and LVI represents a versatile and fast technique for the qualitative and quantitative investigation of artificial sweetener, in the environment with high sensitivity.

## Chemicals and materials

**Fig. 1:**
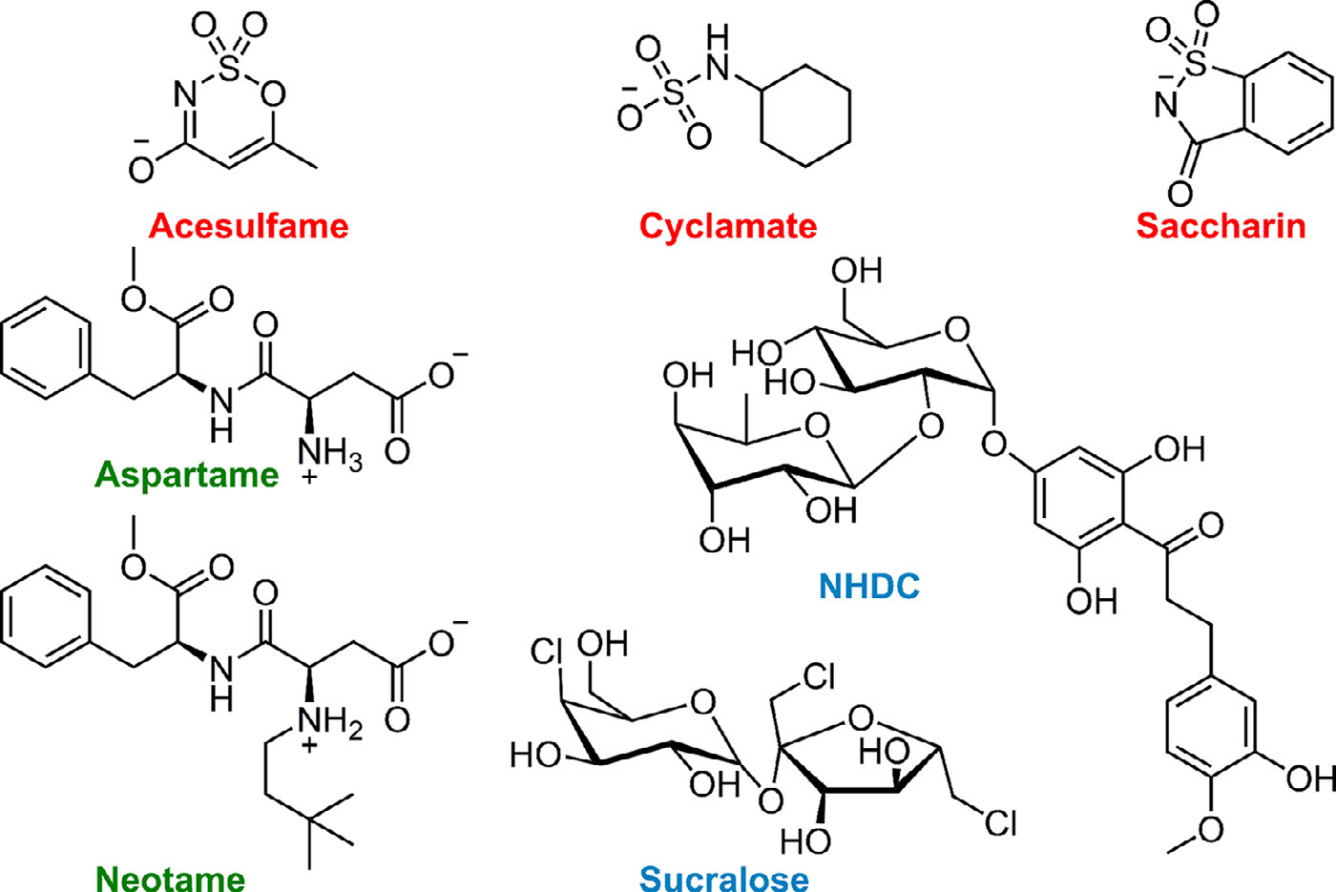
. Structures of the seven artificial sweeteners used in this study. Three highly persistent sulfamates (red: acesulfame, cyclamate and saccharin), two derivates of natural substances (blue: sucralose and NHDC) and two degradable dipeptides (green: aspartame and neotame) were investigated. (For interpretation of the references to color in this figure legend, the reader is referred to the web version of this article.)

For the preparation of eluents, buffers and sample dilution, acetonitrile (ACN, HPLC gradient grade, VWR, Darmstadt, Germany), ammonium formate (≥99.0%, LC-MS Ultra, Sigma Aldrich, Steinheim, Germany), formic acid (≥98%, LC-MS grade, Sigma Aldrich), ammonium acetate (≥99.99% LCMS Ultra, Sigma Aldrich), acetic acid (≥99.99%, Sigma Aldrich) and distilled water from an Aquatron A4000D system were used (Barloworld Scientific, Nemours, France). Analytical standards of acesulfame potassium (>98.0%, TCI, Eschborn, Germany), sodium cyclamate (99.0%, VWR), saccharin (>99.0%, TCI), aspartame (>98.0%, TCI), neotame (98%, J&K Scientific, Pforzheim, Germany), neohesperidin dihydrochalcone (NHDC, >98.0%, TCI) and sucralose (>98.0%, TCI) were used in 50 µM concentration for method development. The three classes of artificial sweeteners and their varying structural features are shown in Fig. 1.

## LC-MS/MS analysis

The analysis of artificial sweeteners was performed on a 1100 series HPLC system (Agilent Technologies, Santa Clara, CA, USA) equipped with a multidraw upgrade kit (400 µL extension, Agilent) and a 100 µL sample loop. The mixed-mode separation was conducted in reversed-phase chromatography mode on a Kaseisorb LC ODS-SAX Super column (150 × 2.0 mm, 3.0 µm; TCI) at 40 °C and a flow rate of 0.25 mL min^1^. The stationary phase comprised C18-alkyl chains with an embedded anion exchange group. The injection volume was 10 µL and was subsequently increased to large volume injections of 500 µL. Mass spectrometric detection in positive/negative switching mode was performed using an EVOQ Elite™ triple quadrupole-mass spectrometer (Bruker, Bremen, Germany). The heated electrospray ionization source operated at ±4000 V with cone and heated probe temperature at 350 °C with a cone gas flow of 20 units, a probe gas flow of 40 units and a nebulizer gas flow of 60 units. For the quantification of artificial sweeteners, an optimization of the respective multiple reaction monitoring (MRM) experiment was conducted; the results are shown in Table 1. Compass Hystar 4.0 and MS workstation (both Bruker) were used for the LC-MS instrument control, data acquisition and data evaluation.

**Table 1:** Optimized parameters for precursor ion selection (Q1), product ions (Q3) and the respective Q2 collision energy for the MRM experiments regarding quantification of seven artificial sweeteners using electrospray ionization in negative mode (top) and positive mode (bottom). The mass transitions used for quantification are shown in bold. Please note that for cyclamate just one intense MS/MS fragment could be obtained and thus, only one MRM transition was used.

Analyte (negative mode)	Q1 [M-H]^−^ [*m/z*]	Q3 [M-H]^−^ [*m/z*]	Collision energy [V]
Acesulfame	162.2	**82.1**/78.1/40.4	−11/−23/−18
Cyclamate	178.2	**80.1**	−23
Saccharin	182.2	**106.0**/42.3	−17/−20
Sucralose	441.0 (+formate)	**394.9**/358.9	−6/−9
NHDC	611.2	**302.9**/165.9/125.0	−36/−54/−42


Analyte (positive mode)	Q1 [M + H]^+^ [*m/z*]	Q3 [M + H]^+^ [*m/z*]	Collision energy [V]
Aspartame	295.0	**120.1**/180.0/235.0	+26/+13/+11
Neotame	379.0	**172.0**/319.1/120.1	+21/+16/+34

**Fig. 2:**
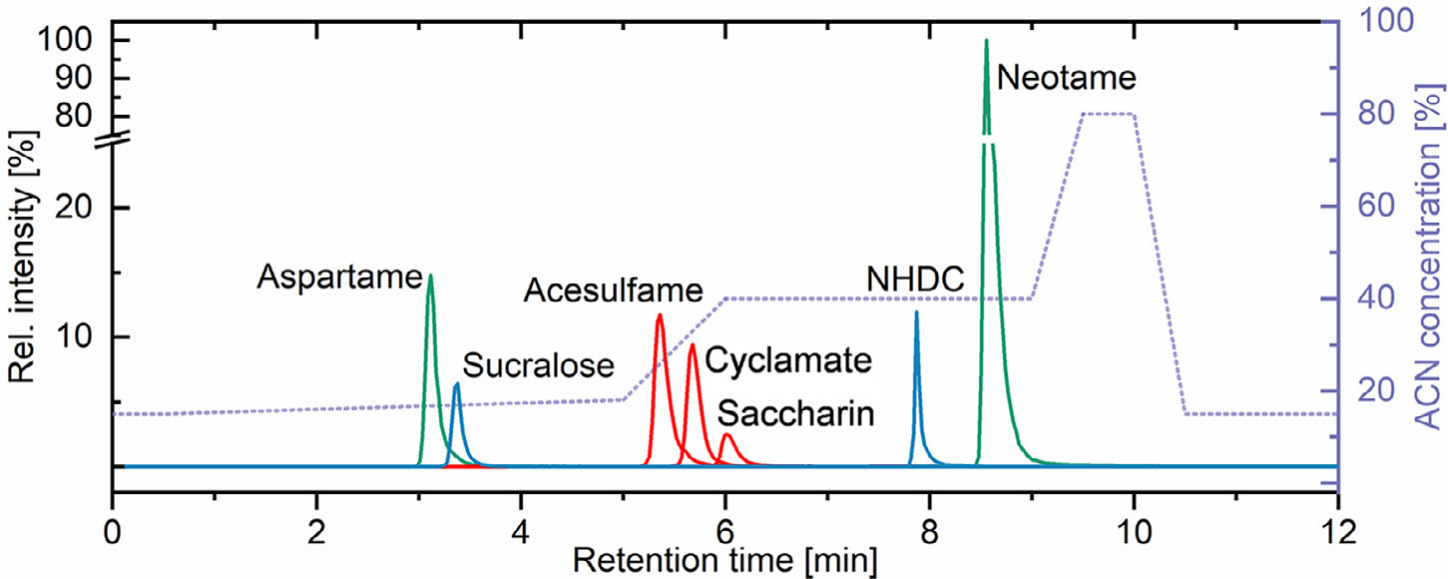
Optimized separation of seven artificial sweeteners on an ODS-SAX mixed-mode stationary phase. The eluent starting condition was 85:15 (v/v) 20 mM ammonium formate at pH 3.5 and ACN. The applied gradient is indicated.

The optimization of the mixed-mode chromatography comprised (i) variation of the buffer composition, (ii) adjustment of the pH and (iii) gradient improvement for chromatographic resolution and reduced separation time. Increasing buffer concentrations from 5 to 20 mM ammonium formate at pH 3.5 resulted in a reduced retention time for the three sulfamates acesulfame, cyclamate and saccharin. The retention time of the further analytes remained unaffected. Consequently, the higher salt concentration leads to reduced interactions between negatively charged sulfamates and the anion exchange group of the stationary phase. Moreover, peak shapes were improved due to the suppression of the anion exchange mechanism (in particular for NHDC). Subsequently, the impact of three different pH (2.7, 3.5, 4.3) was investigated in a close pH range to maintain the positive/neutral charge of the dipeptides aspartame and neotame. The latter showed the overall highest retention, which is traced back to ion exchange mechanism in addition to hydrophobic interactions. With respect to decrease the analysis time, less retention was desired. However, the variation of the pH did not affect neither the anion exchange groups of the stationary phase nor the analytes to a significant extent. Only minor changes like an increased retention for dipeptides was observed for higher pH, correlating with the net charge of these molecules in the eluent (modifying from positive to neutral, slightly improving C18 interaction). Considering the chromatographic results from (i) and (ii), the gradient optimization was conducted at pH 3.5 and starting with 85/15 (v/v) 20 mM ammonium formate and ACN. The final two-step gradient started with 15% ACN from 0 to 5 min and increased in concentration to 40% and 80% ACN as shown in Fig. 2.

## Large volume injection

The large volume injection (LVI) is a valuable alternative to preconcentration techniques for improved sensitivity [10]. LVI are defined having an injection volume >10% of the column void volume [11]. In case of analytes covering a wide polarity range, the LVI can outperform issues of analyte loss and poor recovery rates after offline-solid phase extractions. For the implementation of an LVI, the consideration of the increased sample loop volume and starting conditions with a minimum of mobile phase elution strength was required. Furthermore, the six-port valve remained in the “inject” position after sample injection to flush the loop during the chromatographic run, reducing the possibility of carry-over effects without the need of a further pump. Consequently, the response time for the gradient elution was delayed by 2 min (500 µL loop, 0.25 mL min^1^ flow rate).

**Fig. 3:**
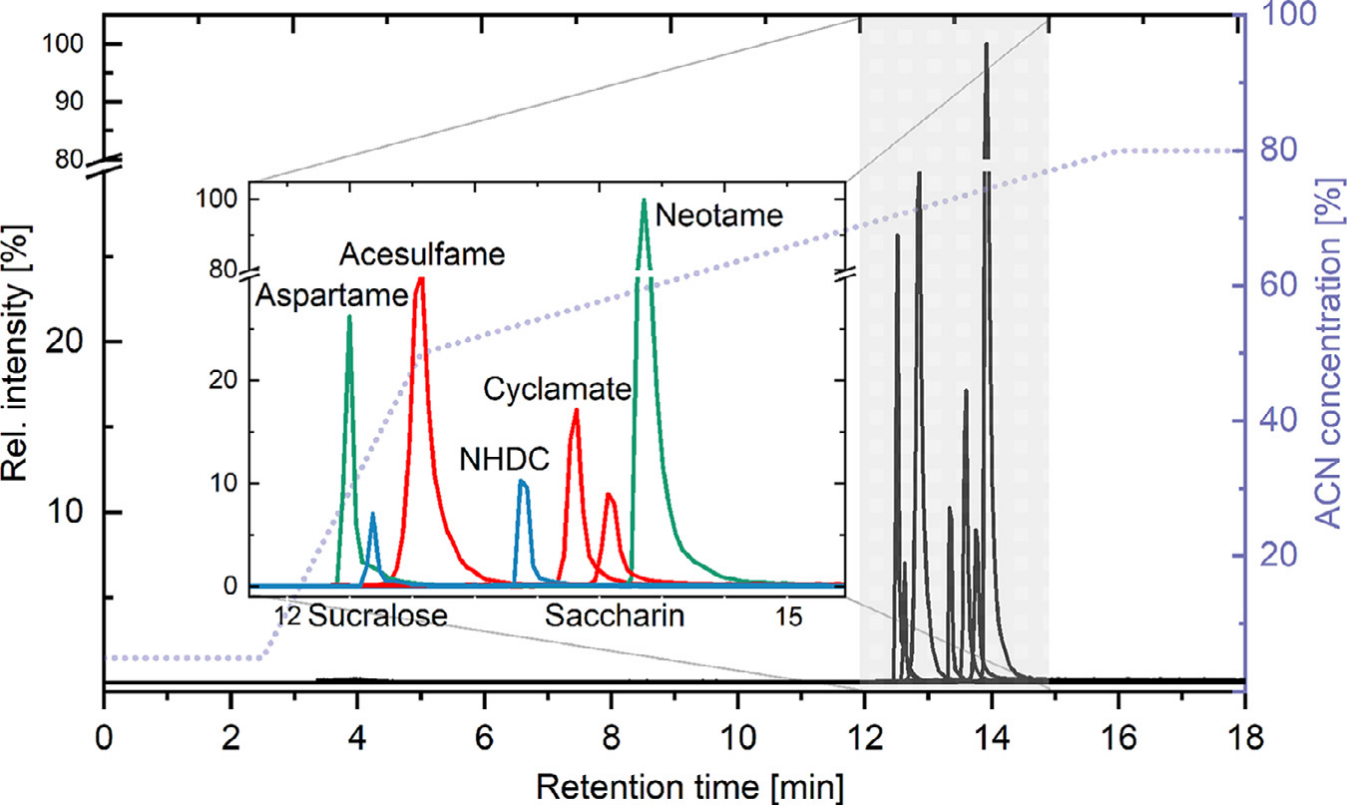
Modified chromatography for LVI of 500 µL sample. The excerpt shows the maintained chromatographic separation of artificial sweeteners following LVI.

The adaption of the starting conditions to 5% ACN led to a refocusing and concentration of the compounds at the head of the analytical column. Overall, a reasonable increase in separation time and a reduced chromatographic resolution (analyte window of only 2.5 min) were observed after the injection of 500 µL. Nonetheless, all artificial sweeteners were successfully separated. The final gradient conditions for LVI were 5% ACN constant for 3 min followed by a first gradient step to 60% within 2.5 min and a second step to 80% ACN within 11 min (see Fig. 3, dotted line). Hence, the application of LVI increased the analysis time by ≈5 min, while the injection volume was 50x higher. Furthermore, the organic content in the effluent was increased from 40% to 70–80% after the method modification improving nebulization and ionization efficiency of the electrospray ionization source.

**Fig. 4:**
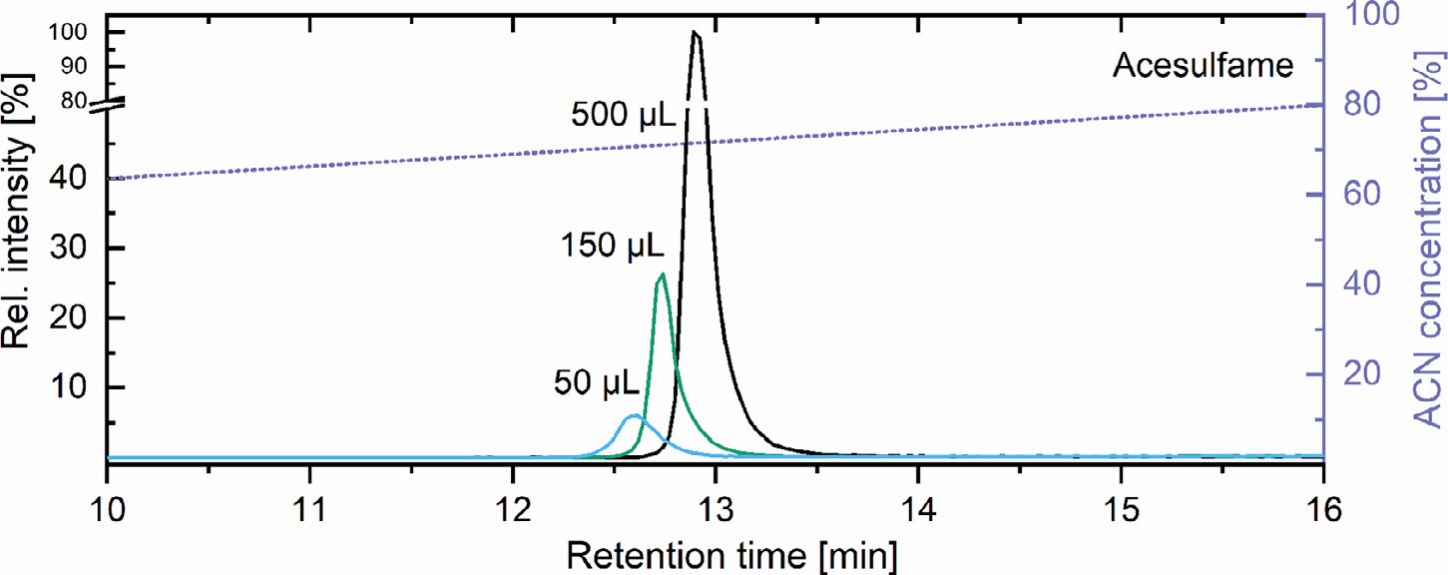
Impact of the increasing injection volumes on the chromatography. Acesulfame is shown as a representative having the broadest initial peak shape at 10 µL injection volume.

According to the applied flow rate, the transfer time from the sample loop to the analytical column was two minutes. Therefore, a possible peak broadening due to diffusion and inefficient column head refocusing was investigated, and for all seven artificial sweeteners the signals from 50 to 500 µL in 50 µL steps were monitored. The peaks of acesulfame at varying injection volumes are shown in Fig. 4. This compound eluted in the center of the retention window and showed the overall broadest signal within the equimolar concentrated standards. For the three representative injection volumes (50, 150 and 500 µL), only a slight shift in retention time and minor peak broadening was observed. Hence, the column head refocusing was successful and the chromatographic resolution was maintained. Thus, a significant improvement of detection limits was achieved. Consequently, the sample preparation was simplified to a membrane filtration (0.2 µm polytetrafluoroethylene (PTFE)). Please note, that due to the marginal sample preparation, a faster degradation of the column performance due to the increased matrix load can be assumed and has to be balanced against the time-saving sample preparation (e.g., in terms of costs). After method development and the described sensitivity and reproducibility testing, a proof of concept study was performed on >40 samples from surface waters (data not shown here). All samples were measured three times after membrane filtration and no adverse effects could be determined (e.g., retention time shifts, deteriorated peak shapes).

## Sensitivity and reproducibility

The applicability of LVI for all seven artificial sweeteners was monitored from 50 to 500 µL. The obtained correlation between peak area and injected volume showed good linearity (Table 2, LVI_corr_) with relative standard deviations below 7% for six out of seven artificial sweeteners. Regarding the multiple injections necessary for higher volumes, the deviations were remarkably low. Hence, LVI is scalable in this injection volume range to improve limits of detection (LODs) and limits of quantification (LOQs). The latter are shown in the middle of Table 2 for an injection volume of 500 µL. LODs and LOQs were determined empirically due to low noise signals in some MRM transitions and are in the low and sub-ng L^1^ range. For the obtained values at least a S/N ratio of 3 and 10 was ensured, respectively. The two columns on the right in Table 2 depict the reproducibility of 500 µL injections (*n* = 9, *c* = 500 pM). Overall, the relative standard deviations were below 8% and only acesulfame exhibited a higher value (20%), possibly due to the broader peak and integration inaccuracies. This assumption gets endorsed by low relative standard deviations for aspartame (5%) and neotame (4%), both showing narrow signals. The presented combination LVI and mixed-mode chromatography is a valuable tool for reliable ultra-trace analysis of artificial sweeteners in surface water samples without elaborate sample preparation steps. The incorporation of stable-isotope labeled standards could improve reproducibility and also could compensate for potential matrix effects affecting quantification.

**Table 2:** Comprehensive overview of LVI implementation (first column), empirically determined limits of detection and quantification (second column) and obtained parameters for the reproducibility of 500 µL injections (third column).

	LVI_corr_ [-]	σ [-]	Rel. σ [%]	LOD [ng L^−1^]	LOQ [ng L^−1^]	σ [-]	Rel. σ [%]
Acesulfame	0.997	850	3.5	0.2	40.3	2.39 × 10^6^	19.8
Cyclamate	0.997	423	5.9	0.1	40.2	1.43 × 10^5^	5.2
Saccharin	0.990	308	5.6	0.2	36.6	1.12 × 10^5^	6.3
Aspartame	0.991	421	6.2	0.6	5.9	1.39 × 10^5^	5.0
Neotame	0.992	1892	6.4	0.4	75.7	9.10 × 10^5^	4.2
Sucralose	0.999	97	5.2	0.4	79.5	4.13 × 10^4^	4.6
NHDC	0.958	415	25.0	1.2	122.5	1.14 × 10^5^	7.9

## Declaration of Competing Interest

The authors declare that they have no known competing financial interests or personal relationships that could have appeared to influence the work reported in this paper.

## References

[1] Lin H., Oturan N., Wu J., Sharma V.K., Zhang H., Oturan M.A. (2017). Removal of artificial sweetener aspartame from aqueous media by electrochemical advanced oxidation processes. Chemosphere.

[2] Scheurer M., Schmutz B., Happel O., Brauch H.J., Wülser R., Storck F.R. (2014). Transformation of the artificial sweetener acesulfame by UV light. Sci. Total Environ..

[3] Arbeláez P., Borrull F., Pocurull E., Marcé R.M. (2015). Determination of high-intensity sweeteners in river water and wastewater by solid-phase extraction and liquid chromatography-tandem mass spectrometry. J. Chromatogr. A.

[4] Lange F.T., Scheurer M., Brauch H.J. (2012). Artificial sweeteners-a recently recognized class of emerging environmental contaminants: a review. Anal. Bioanal. Chem..

[5] Kubica P.P., Namieśnik J., Wasik A. (2015). Determination of eight artificial sweeteners and common stevia rebaudiana glycosides in non-alcoholic and alcoholic beverages by reversed-phase liquid chromatography coupled with tandem mass spectrometry. Anal. Bioanal. Chem..

[6] Henschel J., Schriewer A., Hayen H., Jiang W. (2016). Analysis of artificial sweeteners by HILIC-MS method. LC GC Eur..

[7] Ribbers K., Breuer L., Düring R.A. (2019). Detection of artificial sweeteners and iodinated X-ray contrast media in wastewater via LC-MS/MS and their potential use as anthropogenic tracers in flowing waters. Chemosphere.

[8] Ruta J., Rudaz S., McCalley D.V., Veuthey J.L., Guillarme D. (2010). A systematic investigation of the effect of sample diluent on peak shape in hydrophilic interaction liquid chromatography. J. Chromatogr. A.

[9] Cárdenas-Soracá D.M., Singh V., Nazdrajić E., Vasiljević T., Grandy J.J., Pawliszyn J. (2020). Development of thin-film solid-phase microextraction coating and method for determination of artificial sweeteners in surface waters. Talanta.

[10] Wu M., Qian Y., Boyd J.M., Hrudey S.E., Le X.C., Li X.F. (2014). Direct large volume injection ultra-high performance liquid chromatography-tandem mass spectrometry determination of artificial sweeteners sucralose and acesulfame in well water. J. Chromatogr. A.

[11] Busetti F., Backe W.J., Bendixen N., Maier U., Place B., Giger W., Field J.A. (2012). Trace analysis of environmental matrices by large-volume injection and liquid chromatography-mass spectrometry. Anal. Bioanal. Chem..

